# Management of community acquired pneumonia by Family Physicians

**DOI:** 10.12669/pjms.334.12577

**Published:** 2017

**Authors:** Saima Akhter, Nadeem Rizvi, Sajeer Bhura, Usman Ali Warraich

**Affiliations:** 1 Dr. Saima Akhter, Ziauddin Medical University and Hospital, Karachi, Pakistan; 2 Prof. Nadeem Rizvi, Chest Medicine, Jinnah Post Graduate Medical Centre (JPMC), Karachi, Pakistan; 3 Dr. Sajeer Bhura, Chest Medicine, Jinnah Post Graduate Medical Centre (JPMC), Karachi, Pakistan; 4 Mr. Usman Ali Warraich, Iqra University, Karachi, Pakistan

**Keywords:** Family Physicians, Community Acquired Pneumonia, Quinolones

## Abstract

**Background and Objective:**

Community Acquired Pneumonia (CAP)is a major burden on health systemwith significant mortality and morbidity. Family Physicians(FPs)can play important role. To determine management strategies and prescription of FPs regarding CAP.

**Methods:**

A multicenter cross sectional survey was done in 10 cities of Pakistan from November 2014 to January 2015. Self-administered questionnaire was filled by 110 Family Physicians.

**Results:**

Of total 71% of FPs reported to work in high prevalence areas for respiratory ailments. Only 32% of FPs used PSI and 34% CURB 65 for assessment of severity. It was alarming to note that only 58% of FPs treats severe pneumonia with Intravenous antibiotics while rests were comfortable with oral route. The overall use of quinolones to treat CAP, irrespective of severity, in combination or as single agent was > 60%. Duration of antibiotics for severe pneumonia was sub optimal (<10 days). Only 52.8% patients came back for follow-up so true outcome cannot be anticipated.

**Conclusion:**

Major deficiencies were treatment of severe pneumonia in community, inappropriate use of quinolones and poor knowledge of recent guidelines. This can lead to emergence of resistant bacteria and high mortality and morbidity.

***List of Abbreviations:* FPs:** Family Physicians, **CAP:** Community Acquired Pneumonia.

## INTRODUCTION

Community acquired pneumonia (CAP);lower respiratory tract infection, have high prevalence as well as high mortality and morbidity. Although pneumonia remains a disease taught widely in medical schools and dealt commonly in primary and territory care setups, there is inability to correctly address the disease. Factors accounting for increased mortality include late or missed diagnosis and also inappropriate choice or inadequate duration of antibiotics. In underdeveloped countries other factors like lack of infrastructure, high prevalence of Tuberculosis, unhygienic condition and poverty do contribute to the problem.

The risk of pneumonia increases with age, making it the leading cause of death among elderly.^1^,^2^ In united states alone 600,000 elderly get hospitalization for CAP^3^ and 59,000 suffer death every year.^4^ Although many antibiotics are available, a meta-analysis suggests that the short term mortality in CAP in a hospital or ambulatory setting is 5.1% and it can extend up to 36.5% for ICU patients.^5^ Two recent studies show that CAP is alsoa common infection in the working age population, especially in adults with co-morbidities, where they are estimated to cost $8.5 billion directly and $2.1 billion indirectly in USA.^6^,^7^ Although data from under developed countries of South Asia and Africa is yet to be collected but the burden is expected to be higher as a vast proportion of population from these countries relies on general practitionersfor their health issues. WHO has described the health profile of Pakistan by high maternal mortality, high infant and child mortality, high population growth rate, and increasing burden of communicable and non-communicable diseases.^8^ Of the communicable diseases, community acquired pneumonia deserves special attention.

Although since its advent, Pakistani population had limited access to proper health care, but the number of medical graduates had been increasing since then, with a dramatic rise seen from 1300 in 1977 to 3800 in 1988.^9^ With respect to population, currently the doctor to patient ratio in Pakistan is 1:1300 thatincreased from a baseline of 1:60,000 in 1947.^10^,^11^ Even with doctors graduating in such large numbers, the scarcity is still not addressed and there is an uneven distribution of health services among urban and rural areas.^12^ There are other factors contributing to this situation, like allocation of only 10 hours of overall curriculum time for teaching of general practice in the whole MBBS curriculum, by Pakistan Medical & Dental Council^13^ lack of organized system for postgraduate training or certification courses for primary care physicians and inadequate refresher courses like CMEs for Family Physicians (FPs).

Family Physicians act as backbone of any health care system and are usually the first reference for patients, seeking medical attention for symptoms ranging from simpleflu to a complex diagnosis that require for multidisciplinary approach. Family Physicians can play a very important role in management of CAP by immediate diagnosis, appropriate antibiotic regime and by identifying risk factors. The available evidence also suggests that proper utilization of primary care physicians can successfully reduce unnecessary hospital admissions and consequently national health costs.^14^ With all such aspects this survey was designed to assess the management of CAP among general practitioners across Pakistan.

Our objective was to determine, at a community level, general physician’s knowledge about community acquired pneumonia and to describe their prescription pattern and management strategies.

## METHODS

A multi-centric cross-sectional survey was conducted in 10 different cities of Pakistan. According to demographic parameters and population cities were divided into six large cities and four small towns. Selection of cities was made in a way to cover all major urban areas across the country. The survey was conducted for three months between November 2014 and January 2015. A list of registered Family Physicians is available on official website of Pakistan Medical and Dental Council (PMDC). A Clinical Researchorganization (CRO) was appointed for collection of data and 110 general practitioners were invited to participate in this prospective survey. Equal number of physicians from all socioeconomic areas of the selected cities was selected. Ethical Approval was taken from Pakistan Chest Society.

Only certified Family Physicians with minimum of three years’ experience were included. All these FPs worked in basic health unit, non-government organizations (NGOs) or run their private clinics. These physicians spent at least 70% of their practicing time in patient’s health management and were not engaged in any research project, administration or University teaching jobs.

The questionnaire was designed by two senior pulmonologist and was translated in both Urdu and English to avoid any language barrier in understanding and answering the questions. The investigators, non-medical Professional from CRO, approached the Family Physicians at their clinics and the questionnaires were administered after brief description of survey and nature of questions. They were requested to complete the Performa at the spot and anonymity of responders was maintained. The questionnaire was designed to assess following parameters:

1. Actual load of patients with respiratory ailment on Family Physicians.
2. Confidence among Family Physicians regarding diagnostic skills.
3. Knowledge regarding pathogenesis and current treatment options of CAP.
4. Awareness of referral criteria and complications of CAP.

The data collected was analyzed using SPSS version 17. Statistical significance was taken at p value less than 0.05.

## RESULTS

One hundred and ten Family Physicians took part in the study, 70 were from large cities and 40 from small towns. Out of total population 71% reported to working in areas with high prevalence for respiratory tract infections. Average number of patients seen by these physicians, in a month was 1800 in large cities and 1150 in small towns and 28% of these patients were with respiratory ailment. The number of patient seen by these physicians in a month time showed their popularity in their respective areas and also impact of their decisions on health of community.

Regarding diagnostic skills only 5% consider physical signs reliable to make confident diagnosis, remaining prefer to get Chest X-Ray and hematological evidence before considering the diagnosis of CAP. Only 56% of Physicians consider new onset cough with fever as most frequent symptoms of pneumonia while rest consider shortness of breath as an important symptom of pneumonia. Streptococcal pneumonia was considered to be most common causative agent in community acquired pneumonia by 54.5% while 22% and 8% considered mycoplasma and legionella as most frequent organism respectively but these figures did not reflect in their pharmacotherapy prescription attitude.

Regarding severity tools 32.1%, 29.6% and 34.6%of physicians claimed to use PSI, BORD and CURB 65 respectively to assess the severity of disease. Physicians reported the use of antibiotics in 85% of people who came in their clinics with respiratory ailments. In mild pneumonia the antibiotic of choice was macrolide (35.5%) followed by amoxicillin (33.6%) and quinolones (9%). The combination therapy of penicillin and macrolide was used by 26% of FPs. In moderate cases the antibiotic class more frequently used as monotherapy (29%) was levofloxacin (quinolones) and the most frequent combination was 3^rd^ generation cephalosporin with macrolide (28.2%) while 20% use combination of quinolones with different other antibiotics. The overall use of quinolones to treat CAP, irrespective of severity, in combination or as single agent was more than 60%. The most frequent duration of antibiotics prescribed was 7-10days regardless of severity mentioned before. Only 17.4% of physicians gave 14 days course of antibiotics in severe pneumonia.

The alarming feature was that Family Physicians start treatment in community even if they agree with severe nature of pneumonia and then only 58% of them started antibiotics via Injectable route while rest were comfortable starting antibiotics via oral route. There was also overall reluctance for referral to territory hospital or specialist.

**Fig. 1:**
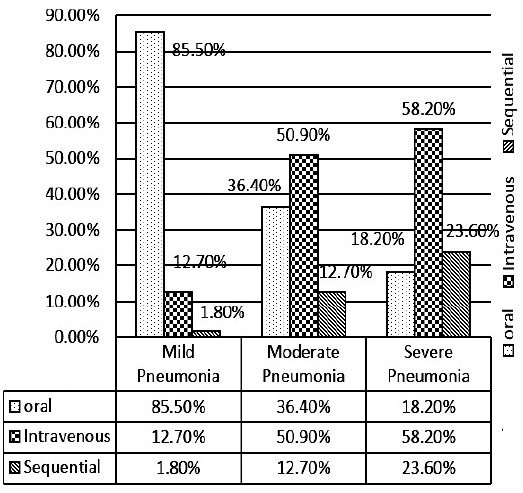
Route of antibiotics given to patients according to severity. Sequential means family Physicians start patients on oral antibiotics and then switch to intravenous antibiotics after seeing worsening in symptoms.

Route of antibiotics given to patients according to severity. Sequential means Family Physicians start patients on oral antibiotics and then switch to intravenous antibiotics after seeing worsening in symptoms is shown in Fig. 1

Only 18% of Family Physicians use good lung penetration and 43% use MIC (Minimum Inhibitory Concentration) as criteria for selection of antibiotics while51% consider cost and easy availability of antibiotics. In respect to awareness of local guidelines of pneumonia 70% claim they heard about the guidelines but only 30% said they actually read it.

Average percentage of patients who came for follow ups to Family Physicians after first evaluation was 52.08%. During follow up visits 38% of FPS used clinical improvement including resolution of fever and cough to assess the treatment response and consider it enough to label patient cured while majority feel comfortable to repeat Chest X-ray and leukocyte count. Regarding pneumonia related complication 38% consideredPara-pneumonia effusion if patient is not responding to treatment while 15% considered abscess as most likely cause.

## DISCUSSION

In present study wefound that the basic knowledge for the spectrum of pathogens and mode of antibiotics was sufficient, but the duration and selection of antibioticswereweak areasamong FPs. Data indicated that inappropriate quinolones’use mask the diagnosis of Tuberculosis^15^ and consequently delay its treatment. Family Physicians practicing in areas where TB prevalence reaches 342/100,000,^16^ over usage of quinolones makes the population vulnerable to further spread of disease and thus makes it difficult to control.

Since Pakistan shares the history with India, Bangladesh and Nepal, the basic problems in the region are still same including high prevalence of tuberculosis, lack of infrastructural and inadequate audits in primary health care system. There is currently no data from South Asia representing level of care for patients with pneumonia by Family Physicians in community. Inpatient mortality due to pneumonia is reported in India in a small study as 11% where immune-suppressed patients and those requiring ICU treatment were excluded.^17^ Though this is similar to what a study from Pakistan reports,^18^ but these numbers do not represent the whole region and more studies are required.

It was also found that doctors were uncertain as how to prioritize the patients according to their disease severity. Though many of the FPs claimedto useseverity scores for triage of the patient but their reluctance for referring the patient to tertiary care facility, treating severe pneumonia as outpatient and preferring oral over Injectable antibiotics, even for severe pneumonia, were matter of serious concern. All these hard facts lead us to two conclusions, first general practitioners didn’t receive the required training to deal with diseases of this magnitude and secondly their detachment from academic activities for a long time has kept them away from current treatment protocols.^19^

### Limitation

This survey covers only urban areas of Pakistan. There is uneven distribution of health services and population in urban versus rural areas, 70% of the population resides in villages whereas 85% of the physicians and 95% of hospital beds are concentrated in urban areas of Pakistan.^12^ The flaws, however, noted in this survey is a core presentation of what cities and towns of Pakistan offer to CAP patient, and it can be easily be anticipated as how things would be working out in rural parts of Pakistan.

### Suggestions

We would like to make following suggestions for the sake of improvement ofprimary healthcare system:

- A proper healthcare infrastructure should be established consisting of Basic Healthcare Units spread throughout every town and village.
- A postgraduate training in Family Medicine should be made necessary with specific attention on management of common disease conditions.
- Regular seminars and continued medical education (CME) should be arranged for Family Physicians from urban as well as rural areas in teaching universities to update their knowledge and improve their practice according to latest guidelines.
- Display of clinical tools like PSI and CURB-65 in form of posters should be encouraged in the clinics, as it would serve as a reminder for doctor.
- Although local guidelines are present but these guidelines rely heavily on foreign data, studies should be carried out in this region so that ground realities are better realized and dealt with.
- Communications between the hospitals should be encouraged as it would help doctors in referring patients to a better place with better facility
- Health indicators should be used on annual basis to continuously assess the performance of all public and private healthcare setup, and should be compared with previous years and with others in the same area.

If the principals of better treatment is followed in cases of CAP then it is expected that major chunk of health budget will be utilized in treating more serious diseases like Tuberculosis and cancer. A decrease in antibiotic resistance will also save the grants that can be allocated for intense research into new ways of overcoming resistance and it also increase patient compliance and reduce side-effects. The most important aspect that deserves mention is decreasing load on tertiary care hospitals saving space, time and resources for other serious problems that need attention.

## CONCLUSION

Several deficiencies were seen in management of CAP and the major problems encountered were trend of treating severe pneumonia in community, inappropriate use of quinolones, poor knowledge of recent guidelines and inadequate questioningabout co-morbid conditions, that in turn can lead to high mortality and morbidity and emergence of resistant bacteria as cause of CAP.
